# A specific combination of laboratory data is associated with overweight lungs in patients with COVID-19 pneumonia at hospital admission: secondary cross-sectional analysis of a randomized clinical trial

**DOI:** 10.3389/fmed.2023.1137784

**Published:** 2023-05-16

**Authors:** Pedro L. Silva, Fernanda F. Cruz, Camila M. Martins, Jacob Herrmann, Sarah E. Gerard, Yi Xin, Maurizio Cereda, Lorenzo Ball, Paolo Pelosi, Patricia R. M. Rocco

**Affiliations:** ^1^Laboratory of Pulmonary Investigation, Institute of Biophysics Carlos Chagas Filho, Federal University of Rio de Janeiro, Rio de Janeiro, Brazil; ^2^AAC&T Research Consulting LTDA, Curitiba, Brazil; ^3^Roy J. Carver Department of Biomedical Engineering, University of Iowa, Iowa City, IA, United States; ^4^Department of Radiology, Perelman School of Medicine, University of Pennsylvania, Philadelphia, PA, United States; ^5^Department of Anesthesia, Critical Care and Pain Medicine, Massachusetts General Hospital, Boston, MA, United States; ^6^Department of Surgical Sciences and Integrated Diagnostics (DISC), University of Genoa, Genoa, Italy; ^7^Anesthesia and Intensive Care, San Martino Policlinico Hospital, IRCCS for Oncology and Neurosciences, Genoa, Italy

**Keywords:** COVID-19, computed tomography, lung weight, biomarkers, leukocytes, receiver operating curve

## Abstract

**Background:**

Lung weight may be measured with quantitative chest computed tomography (CT) in patients with COVID-19 to characterize the severity of pulmonary edema and assess prognosis. However, this quantitative analysis is often not accessible, which led to the hypothesis that specific laboratory data may help identify overweight lungs.

**Methods:**

This cross-sectional study was a secondary analysis of data from SARITA2, a randomized clinical trial comparing nitazoxanide and placebo in patients with COVID-19 pneumonia. Adult patients (≥18 years) requiring supplemental oxygen due to COVID-19 pneumonia were enrolled between April 20 and October 15, 2020, in 19 hospitals in Brazil. The weight of the lungs as well as laboratory data [hemoglobin, leukocytes, neutrophils, lymphocytes, C-reactive protein, D-dimer, lactate dehydrogenase (LDH), and ferritin] and 47 additional specific blood biomarkers were assessed.

**Results:**

Ninety-three patients were included in the study: 46 patients presented with underweight lungs (defined by ≤0% of excess lung weight) and 47 patients presented with overweight lungs (>0% of excess lung weight). Leukocytes, neutrophils, D-dimer, and LDH were higher in patients with overweight lungs. Among the 47 blood biomarkers investigated, interferon alpha 2 protein was higher and leukocyte inhibitory factor was lower in patients with overweight lungs. According to CombiROC analysis, the combinations of D-dimer/LDH/leukocytes, D-dimer/LDH/neutrophils, and D-dimer/LDH/leukocytes/neutrophils achieved the highest area under the curve with the best accuracy to detect overweight lungs.

**Conclusion:**

The combinations of these specific laboratory data: D-dimer/LDH/leukocytes or D-dimer/LDH/neutrophils or D-dimer/LDH/leukocytes/neutrophils were the best predictors of overweight lungs in patients with COVID-19 pneumonia at hospital admission.

**Clinical trial registration:**

Brazilian Registry of Clinical Trials (REBEC) number RBR-88bs9x and ClinicalTrials.gov number NCT04561219.

## Introduction

In patients with COVID-19, lung weight increases as a result of pulmonary edema (1). Patients with excess lung weight measured by quantitative chest computed tomography (CT) are more likely to evolve to a severe form of COVID-19, with greater need for invasive mechanical ventilation and risk of hospital mortality (2). However, to date, there is no study on patients with COVID-19 pneumonia at hospital admission associating overweight lungs with laboratory data, which may thus predict prognosis.

Our group conducted a randomized clinical trial (RCT) comparing nitazoxanide with placebo in patients with COVID-19 hospitalized with pneumonia (3). A secondary cross-sectional analysis was then performed investigating possible associations between clinical parameters, blood laboratory data, and specific biomarkers using quantitative chest CT scans obtained at hospital admission. We hypothesized that specific laboratory data may help identify overweight lungs in patients with COVID-19 pneumonia.

## Methods

### Study design

A secondary cross-sectional analysis of an RCT (3) was performed to assess laboratory and blood markers as well as quantitative chest CT scans at hospital admission (before the enrolled patients were allocated to placebo and intervention groups) to help identify overweight lungs in patients with COVID-19 pneumonia. This study adheres to the Declaration of Helsinki and the Brazilian National Commission for Research Ethics (CAAE:30662420.0.1001.0008), and individual Ethics Committees of all participating sites approved this study. This trial was registered in the Brazilian Registry of Clinical Trials (REBEC) number RBR-88bs9x and ClinicalTrials.gov number NCT04561219. The study description followed the STROBE guidelines (4).

### Patients

This secondary cross-sectional analysis included consecutive patients with COVID-19 pneumonia admitted to 19 hospitals in Brazil from April 20 to October 15, 2020. Inclusion criteria were as follows: adult patients (≥18 years) requiring supplemental oxygen [peripheral oxygen saturation (SpO_2_) <93%], admitted to the hospital with symptoms of COVID-19 associated with chest CT findings suggestive of viral pneumonia or a positive nasopharyngeal swab test for SARS-CoV2 [reverse transcription polymerase chain reaction (RT-PCR)] with laboratory data and blood markers as well as a quantitative chest CT scan (DICOM files; Supplementary Figure 1). The exclusion criteria were as follows: history of severe liver disease, chronic kidney disease defined as an estimated glomerular filtration rate < 30 mL/min/1.73 m^2^, severe heart failure (New York Heart Association class 3 and class 4), severe chronic obstructive pulmonary disease (Global Initiative for Obstructive Lung Disease class 3 and 4), any cancer in the last 5 years, any known autoimmune disease, known allergy to nitazoxanide or its components, nitazoxanide treatment in the last 30 days, clinical suspicion of tuberculosis or bacterial pneumonia.

### Variables

The outcome variable was under- or overweight lungs according to the expected weight, as well as clinical and laboratory variables: age, sex, ethnicity, body mass index (BMI), temperature, respiratory rate, SpO_2_, hemoglobin (Hb), leukocytes, neutrophils, lymphocytes, C-reactive protein, D-dimer, and ferritin.

### Data sources/measurements

#### Demographic, clinical, and laboratory data at hospital admission

Demographic data (age, sex, ethnicity, BMI), clinical parameters, coexisting conditions, concomitant medications, patients’ symptoms, and diagnosis of SARS-CoV2 infection (positive or negative swab test) were collected at hospital admission. Site investigators performed a comprehensive physical examination, recording levels and type of oxygen supplementation and concomitant medications Further clinical data regarding time from symptom onset until randomization, temperature, respiratory rate, peripheral oxygen saturation (SpO_2_) and laboratory data, such as hemoglobin (Hb), white blood cells (leukocytes), neutrophils, lymphocytes, C-reactive protein, D-dimer, lactate dehydrogenase (LDH), and ferritin were also evaluated. Study data were entered directly into electronic case-report forms (REDCap) and clinical trial management system by the site investigator and validated by monitoring staff.

#### Blood biomarkers at hospital admission

At hospital admission, blood samples were taken and complete blood cell counts and the other parameters were analyzed at the local laboratory of each hospital. Cryotubes were labeled with the patient’s unique trial identifier at admission. Blood biomarkers were analyzed using multiplex commercial kits for the detection of 47 human cytokine biomarkers (Bio-Plex Pro Human Cytokine Screening Panel, 48-Plex. 1 × 96-well), which included basic fibroblast growth factor (B-FGF), eotaxin, granulocyte colony-stimulating factor (G-CSF), granulocyte-macrophage colony-stimulating factor (GM-CSF), interferon (IFN)-γ, interleukin (IL)-1β, IL-1ra, IL-1α, IL-2Rα, IL-3, IL-12 (p40), IL-16, IL-2, IL-4, IL-5, IL-6, IL-7, IL-8, IL-9, growth-related oncogene (GRO) alpha, hepatocyte growth factor (HGF), IFN-α2, leukemia inhibitory factor (LIF), monocyte chemotactic protein (MCP)-3, IL-10, IL-12 (p70), IL-13, IL-15, IL-17A, IP-10, MCP-1, monokine induced by IFN-γ (MIG), nerve growth factor (NGF)-β, stem cell factor (SCF), stem cell growth factor (SCGF)-β, stromal cell-derived factor (SDF)-1α, macrophage inflammatory protein (MIP)-1α, MIP-1β, platelet-derived growth factor (PDGF)-BB, regulated upon activation, normal T cell expressed, and secreted (RANTES), tumor necrosis factor (TNF)-α, vascular endothelial growth factor (VEGF), T cell–attracting chemokine (CTACK), macrophage migration inhibitory factor (MIF), TNF-related apoptosis-inducing ligand (TRAIL), IL-18, macrophage colony-stimulating factor (M-CSF), TNF-β.

#### Chest CT analysis

Patients underwent chest CT imaging at Hospital Municipal de Barueri Dr. Francisco Moran (*n* = 16), Hospital Regional de Sorocaba Dr. Adib D Jatene-Bata Branca (*n* = 13), Hospital Geral de São Mateus (*n* = 2), Hospital das Clínicas Luzia de Pinho Melo (*n* = 3) (São Paulo, Brazil), Hospital da Força Aérea do Galeão (*n* = 19), Hospital Central da Aeronáutica (*n* = 11), Hospital Naval Marcilio Dias (*n* = 13) (Rio de Janeiro, Brazil), Hospital das Forças Armadas (*n* = 7) (Brasília, Brazil), Hospital Estadual de Doenças Tropicais Dr. Anuar Auad (*n* = 3) (Goiás, Brazil), Hospital Geral de Fortaleza (*n* = 1) (Ceará, Brazil), Hospital Mater Dei (*n* = 3) (Minas Gerais, Brazil), Complexo do Trabalhador de Curitiba (*n* = 2) (Paraná, Brazil). Chest CT scans were performed during end-inspiratory breath hold using the following available scanners: General Electric, Optima ct66, VCT Lightspeed, and Brightspeed models; Siemens Somaton Definition; Philips Brilliance and Access models; Hitachi Scenaria. A multi-resolution convolutional neural network was used to generate the lung segmentations and it has been quantitatively evaluated on 93 clinical CT scans of COVID-19 subjects (average symmetric surface distance of 0.495 ± 0.309 mm and Dice coefficient of 0.985 ± 0.011). This lung segmentation algorithm has been qualitatively evaluated on several researches and clinical CT scans acquired on different scanners (5). Chest CT imaging was done according to the pre-planned protocol of the RCT. Briefly, the major CT findings were described using international standard nomenclature defined by the Fleischner Society glossary and peer-reviewed literature on viral pneumonia, using terms including ground-glass opacity, crazy-paving pattern, pleural effusion, and consolidation (6, 7).

#### Calculation of excess lung weight

The calculation for excess lung weight was based on a previous study (8) according to the following formula: excess lung weight (%) = observed lung weight (g) − expected lung weight (g)/expected lung weight (g) × 100. The expected lung weight (g) was calculated according to the following formula: expected lung weight (g) = −1806.1 + 1633.7 × height (m). Patients were classified with under- or overweight lungs; underweight lungs were defined as ≤0% and overweight lungs were defined as >0% of excess lung weight.

### Statistical analysis

No formal sample size calculation was performed due to the exploratory nature of the study. All available data according to the inclusion criteria were used. Descriptive analyses of the expected, observed, and excess lung weight were performed initially by absolute and relative frequencies. The demographic, clinical and laboratory parameters were described according to the classification below (underweight) or above (overweight) the expected lung weight, with the mean (standard deviation) or median and interquartile interval. The Shapiro–Wilk test was performed between 2 groups to determine the parametric and non-parametric approach. The Student t test was done for those variables with parametric distribution, and the Mann–Whitney test was used for those with non-parametric distribution. To choose the best cutoff points according to the natural data distribution, sensitivities and specificities of individual blood markers in detecting overweight and underweight lungs, the area under the curve (AUC) was calculated and presented with the 95% confidence interval (CI). CombiROC (9) was used to match different receiver operating characteristic (ROC) curves and identify the best combination to detect overweight lungs. Briefly, the criteria to select blood markers to combine ROC curves were the significance at the univariate analysis, and the percentage of missing data <5%. Since the data distributions are different among the blood markers, they were normalized according to the *rescale* function available in the statistical *scales* package (R environment, R Core Team, 2021 (10)). Once the data were normalized, the Youden criteria were used to choose the best threshold for different combinations. Once the threshold was recognized, all available combinations were compared taking into account both the highest AUC and accuracy to detect overweight lungs. All analyses were considered significant when *p* < 0.05 and the analyses were performed in the R 4.0.4 environment (R Core Team, 2021).

## Results

**Figure 1:**
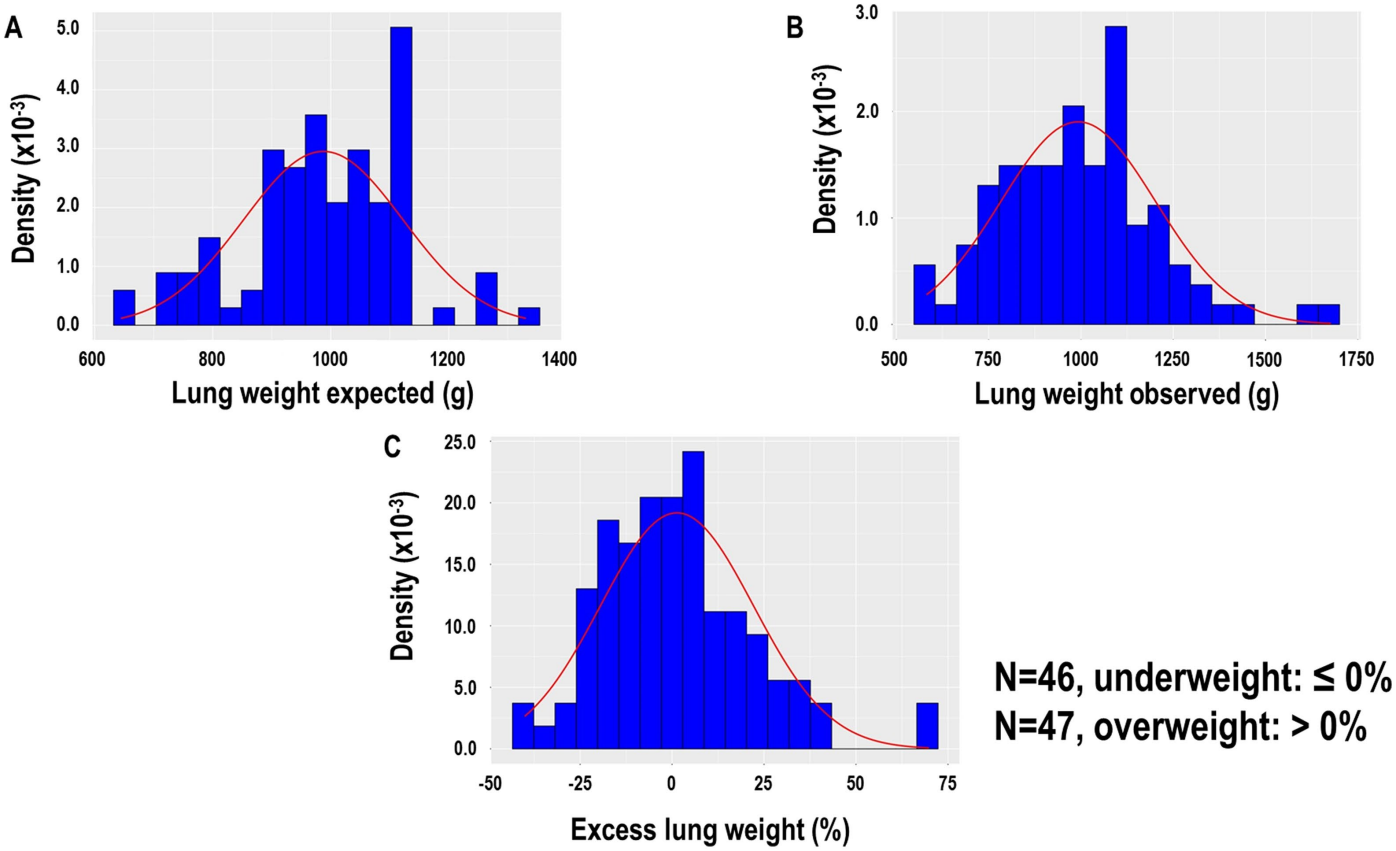
**(A)** Lung weight expected according to the following formula: expected lung weight (G) = −1806.1 + 1633.7 × height (m) of 93 patients. **(B)** Lung weight observed was assumed proportional to the gas versus tissue fraction contained in each voxel, approximating the tissue density as equal to the water density. **(C)** Excess lung weight according to the following formula: excess lung weight (%) = observed lung weight (g) − expected lung weight (g)/expected lung weight (g) × 100.

**Figure 2:**
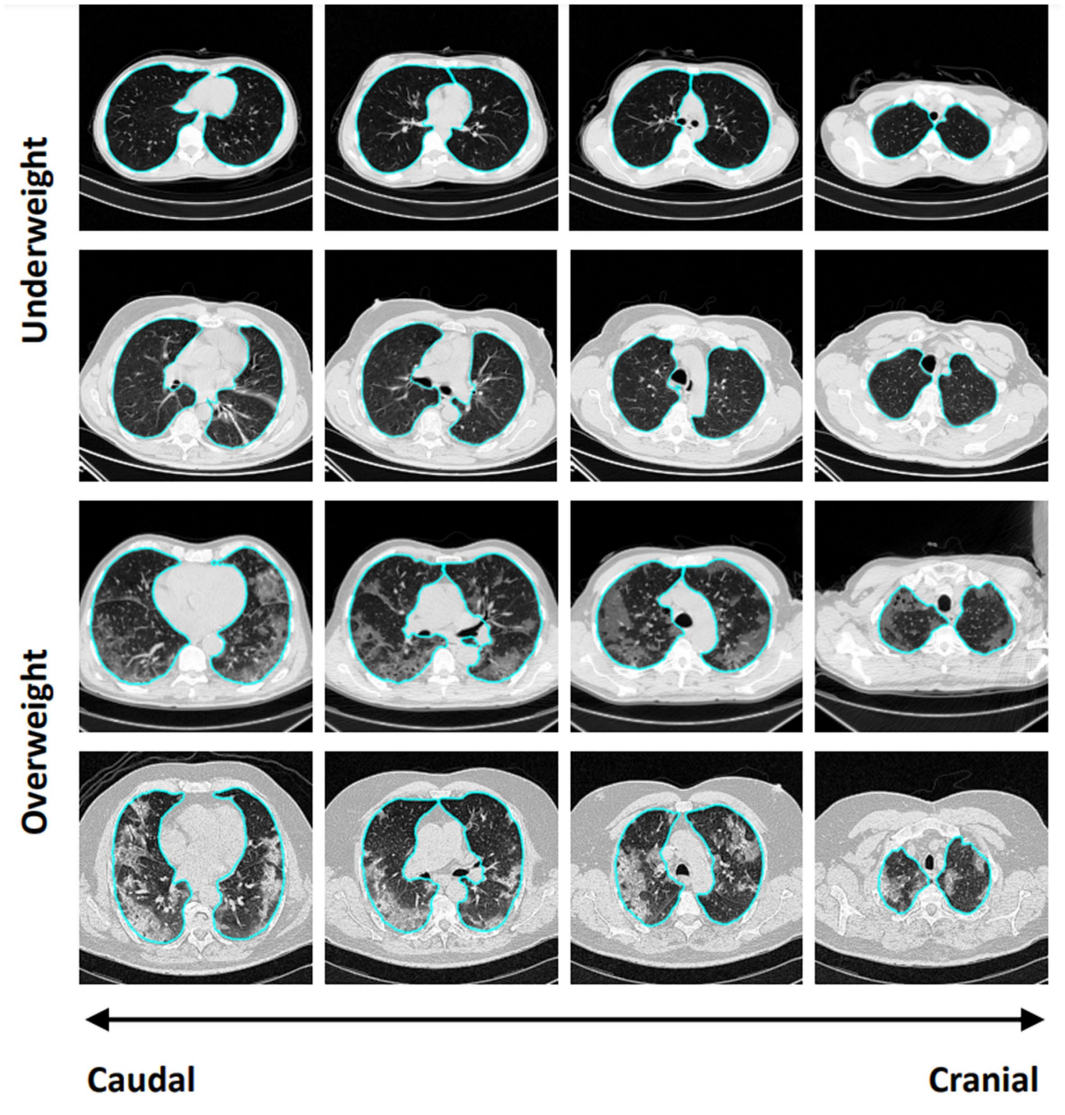
Representative CT images, from cranial to caudal, from two patients with underweight, defined by ≤0% of excess lung weight, and two patients with overweight lungs, defined by >0% of excess lung weight.

Overall, 93 patients were included in the analysis (Supplementary Figure 1). The median [interquartile range (IQR)] of observed, expected, and excess lung weight was 1,005 g (838–1,111 g), 987.5 g (889.5–1085.8 g), and 0.1% (−13.6 to 10.5 g) (Figures 1A–C), respectively. Forty-six patients were classified with underweight lungs, defined by ≤0% of excess lung weight, and 47 patients were classified with overweight lungs, defined by >0% of excess lung weight. Figure 2 shows representative CT images, from cranial to caudal, from two patients with underweight, defined by ≤0% of excess lung weight, and two patients with overweight lungs, defined by >0% of excess lung weight.

No differences were observed with regard to age, sex, ethnicity, and BMI (Supplementary Table 1), as well as coexisting conditions, concomitant medications, patients’ symptoms, and diagnosis of SARS-CoV2 infection (Supplementary Table 2) between patients with underweight and overweight lungs. Leukocytes, neutrophils, D-dimer, and LDH were higher in those patients with overweight lungs (Table 1). One blood marker (VEGF) did not work in the multiplex analysis. Among the 47 blood markers, IFN-α2 was higher and LIF was lower in patients with overweight compared with underweight lungs (Supplementary Table 3).

**Table 1:** Clinical and laboratory data according to lung underweight and overweight.

Clinical and laboratory data	Underweight (*n* = 46)	Overweight (*n* = 47)	*p*-value (Mann–Whitney test)
Time from symptom onset to randomization (days)	6 (5–7)	7 (5–8)	0.351
Temperature (°C)	36.3 (35.8–36.7)	36.4 (36.1–37.0)	0.097
Respiratory rate (breaths/min)	20 (19–24)	20 (18–24)	0.907
SpO_2_ (%)	92 (91–93)	92 (90–92)	0.28
Hb (g/dL)	13.7 (12.7–14.5)	13.5 (12.4–15.1)	0.779
Leukocytes (×10^3^/mL)	6.7 (5.4–7.9)	8.1 (7.4–10.8)	<0.001
Neutrophils (×10^3^/mL)	4.9 (3.6–5.8)	6.2 (5.1–8.3)	<0.001
Lymphocytes (×10^3^/mL)	1.9 (1.4–2.6)	2 (1.4–2.6)	0.451
C-reactive protein (mg/L)	116 (75–149)	133 (96–170)	0.116
D-Dimer (ng/mL)	823 (422–1,338)	1,278 (617–1814)	0.037
Ferritin (mg/L)	459 (250–674)	543 (331–702)	0.305
Lactate dehydrogenase (IU/L)	231 (185–296)	314 (208–364)	<0.001

Values are median (1st and 3rd quartiles). Comparisons were done by Mann–Whitney test (*p* < 0.05). SpO_2_, peripheral oxygen saturation; Hb, hemoglobin.

Among the blood markers, IFN-α2 and LIF had the highest and significant AUC values (Supplementary Table 4). The range of thresholds with the respective sensitivities, specificities, and odds ratios of leukocytes, neutrophils, LDH, D-dimer, IFN-α2 and LIF are shown in Table 2. After applying the CombiROC analysis, the combinations of D-dimer/LDH/leukocytes, D-dimer/LDH/neutrophils, D-dimer/LDH/leukocytes/neutrophils achieved the highest AUC values (0.793, 0.789, 0.792; respectively) with high accuracy (Table 3).

**Table 2:** Thresholds, sensitivities and specificities and odds ratio of laboratory data and blood biomarkers to detect overweight lungs at hospital admission.

Laboratory data and blood biomarkers	AUC (95% CI)	Sensitivity, % (95% CI)	Specificity, % (95% CI)	Odds Ratio (95% CI)	*p*-value
Leukocyte thresholds (×10^3^/mL)	0.722 (0.617–0.827)				
>7,835		57 (57–58)	73 (73–74)	3.6 (1.5–9.1)	0.003
>7,650		66 (65–66)	71 (71–72)	4.6 (1.9–11.7)	<0.001
>7,505		68 (67–68)	67 (66–67)	4.2 (1.8–10.3)	<0.001
>7,150		77 (76–77)	60 (59–60)	4.8 (1.9–12.3)	<0.001
Neutrophil thresholds (×10^3^/mL)	0.713 (0.606–0.820)				
>5,579		62 (61–62)	71 (70–71)	3.8 (1.6–9.3)	0.002
>5,236		68 (67–68)	64 (63–64)	3.7 (1.6–8.9)	0.002
>5,179		72 (72–73)	61 (61–62)	4.1 (1.7–10.2)	0.001
>5,071		79 (78–79)	54 (54–55)	4.3 (1.8–11.3)	0.001
Lactate dehydrogenase thresholds (IU/L)	0.711 (0.601–0.820)				
>301		62 (61–62)	76 (75–76)	4.9 (2.0–12.4)	<0.001
>285		70 (69–70)	71 (71–72)	5.6 (2.3–14.4)	<0.001
>281.5		75 (74–75)	71 (70–71)	6.9 (2.8–18.2)	<0.001
>251		79 (78–79)	58 (57–58)	4.9 (2.0–12.9)	<0.001
D-dimer threshold (ng/mL)	0.626 (0.512–0.741)				
>1,213		53 (53–53)	66 (66–67)	2.2 (0.9–5.4)	0.055
IFN-α2 threshold (IU/mL)	0.764 (0.547–0.982)				
>332.8		55 (54–55)	82 (81–82)	4.8 (0.7–47.3)	0.076
>304.9		64 (63–64)	73 (72–73)	4.8 (0.7–47.3)	0.076
>290.7		82 (81–82)	64 (63–64)	6.8 (1.1–69.2)	0.030
>255.2		82 (81–82)	55 (54–55)	4.8 (0.7–47.3)	0.076
LIF threshold (IU/mL)	0.821 (0.536–1.000)				
<2,793		50 (50–50)	86 (85–86)	0.2 (0.0–12.8)	0.284
<3,485		100 (99–100)	71 (71–72)	–	–
<5,598		100 (99–100)	57 (56–57)	–	–

LIF, leukemia inhibitory factor; IFN-α2, Interferon alpha-2.

**Table 3:** Accuracy of blood laboratory data to detect overweight lungs at hospital admission after CombiROC analysis.

Laboratory data	AUC	SE	SP	ACC	TN	TP	FN	FP	NPV	PPV
D-Dimer	0.738	0.721	0.787	0.756	37	31	12	10	0.755	0.756
LDH	0.714	0.628	0.745	0.689	35	27	16	12	0.686	0.692
Leukocytes	0.709	0.535	0.83	0.689	39	23	20	8	0.661	0.742
Neutrophils	0.627	0.744	0.511	0.622	24	32	11	23	0.686	0.582
D-Dimer/LDH	0.775	0.791	0.787	0.789*	37	34	9	10	0.804	0.773
D-Dimer/leukocytes	0.725	0.512	0.872	0.7	41	22	21	6	0.661	0.786
D-Dimer/neutrophils	0.729	0.767	0.617	0.689	29	33	10	18	0.744	0.647
LDH/leukocytes	0.772	0.767	0.681	0.722	32	33	10	15	0.762	0.688
LDH/neutrophils	0.772	0.791	0.702	0.744	33	34	9	14	0.786	0.708
Leukocytes/neutrophils	0.722	0.698	0.723	0.711	34	30	13	13	0.723	0.698
D-Dimer/LDH/leukocytes	0.793	0.651	0.851	0.756	40	28	15	7	0.727	0.800
D-Dimer/LDH/neutrophils	0.789	0.721	0.745	0.733	35	31	12	12	0.745	0.721
D-Dimer/leukocytes/neutrophils	0.734	0.628	0.745	0.689	35	27	16	12	0.686	0.692
LDH/leukocytes/neutrophils	0.772	0.767	0.702	0.733	33	33	10	14	0.767	0.702
D-Dimer/LDH/leukocytes/neutrophils	0.792	0.651	0.851	0.756	40	28	15	7	0.727	0.800

AUC, area under the curve; SE, sensitivity; SP, specificity; ACC, accuracy; TN, true negative; TP, true positive; FN, false negative; FP, false positive; NPV, negative predictive value; PPV, positive predictive value; LDH, lactate dehydrogenase.

## Discussion

In patients with COVID-19 pneumonia admitted to the hospital, we found that: (1) lung weight has a wide distribution, from underweight to overweight; (2) leukocytes, neutrophils, D-dimer, and LDH at hospital admission were higher in patients with overweight lungs; (3) the combinations of D-dimer/LDH/leukocytes, D-dimer/LDH/neutrophils, D-dimer/LDH/leukocytes/neutrophils better identified patients with overweight lungs. This is the first study on patients with COVID-19 pneumonia focused on identifying overweight lungs, which may provide a promising prognostic tool when associated with specific laboratory data.

At hospital admission, the lung weight of patients with COVID-19 pneumonia may be below or above the expected value for their height. Those patients with positive excess lung weight measured by chest CT analysis are more likely to develop severe forms of COVID-19, with the need for invasive mechanical ventilation and higher in-hospital mortality (2). Chest CT imaging is essential to gather such information. In 2020, the American College of Radiology did not advise the use of chest CT as a first imaging modality in all patients with COVID-19, but only in hospitalized, symptomatic patients with specific clinical conditions (11). The Fleischner Society identified 3 main scenarios when imaging may be used as a primary diagnostic tool: (1) patients with mild respiratory features consistent with COVID-19 but with risk factors for disease progression; (2) patients with moderate to severe features of COVID-19, regardless of RT-PCR test results; and (3) patients presenting with moderate to severe symptoms within a high prevalence of disease environment and with limited testing resources (12). Our population may represent the intersection between the first and second scenarios addressed by the Fleischner Society. In units with limited chest CT resources, important information about lung weight may be inferred by using clinical and laboratory data as well as blood markers, mainly at hospital admission. Recently, Citu et al. (13) showed significant association of chest CT features of patients with COVID-19 and changes in C-reactive protein, IL-6, and neutrophil to lymphocyte ratio. In the present study, we found that leukocytes, neutrophils, D-dimer, and LDH were higher in patients with overweight lungs; and among 47 specific blood markers studied, only IFN-α2 was higher, whereas LIF was lower, in patients with overweight lungs. In patients with COVID-19, recruitment of leukocytes to the respiratory system is orchestrated by specific trafficking molecules, resulting in various pathologic complications in the lungs (e.g., acute respiratory distress syndrome) and in other organs (14, 15). These conditions are characterized by increased permeability of lung capillaries and the entry of solutes and fluids into the alveolar unit, leading to pulmonary edema and therefore overweight lungs. In our study, ROC analyses showed that leukocyte higher than 7,650 × 10^3^/mL and neutrophil levels higher than 5,579 × 10^3^/mL detected positive cases of overweight lungs with 66 and 62% sensitivity, respectively, and discarding the false-negative cases of overweight lung, with 71% specificity for both variables.

D-dimer is a protein fragment present in the blood resulting from clot degradation, and its levels are significantly increased in the edema fluid of patients with classic acute respiratory distress syndrome (16). Our data suggest that D-dimer levels higher than 1,213 ng/mL detected the real positive cases with overweight lungs with 66% sensitivity, and discarding the false-negative cases of overweight lungs, with 53% specificity. Different from our study, a higher threshold of D-dimer has been reported reflecting greater clinical severity of COVID-19 and duration of invasive mechanical ventilation (17). LDH is found in cardiac and skeletal muscles, liver, kidney, brain, and other tissue cells and released into the bloodstream. A previous study found that LDH levels in patients with both mild and severe COVID-19 were significantly higher than those in healthy individuals (18). We found that LDH levels identified patients with COVID-19 who had overweight lungs at hospital admission when the median number of days from symptom onset to hospital admission was 6–7 days. LDH levels higher than 301 IU/L can be an early marker to detect overweight lungs with 62% sensitivity and 76% specificity.

IFN-α2 is significantly upregulated in the COVID-19+ cohort (19), and it is strongly associated with the IFN transcriptional program in immune cells (20) and a poor prognosis. In the current study, IFN-α2 levels higher than 332.8 IU/L were associated with overweight lungs with 82% sensitivity and 55% specificity. IFN drives lung inflammation, and will likely promote leukocyte migration, increasing alveolar permeability, and ultimately leading to overweight lungs during viral infections (21). LIF facilitates tissue protection during pneumonia by activation of signal transducer and activator of transcription (STAT)-3 in lung epithelial cells, which promotes the expression of tissue-protective genes (22) and, during respiratory syncytial viral infection (23), stimulates lung tissue regeneration and repair. Exogenous LIF administration has been proposed as a therapy to protect the lungs and reduce disease severity caused by SARS-CoV-2 (24). We found that LIF levels lower than 2,739 IU/L were associated with overweight lungs with 50% sensitivity and 86% specificity. LIF levels are indirectly proportional to inflammatory conditions, since they are in line with tissue protection (22, 23).

### Limitations

This study has some limitations that should be pointed out. First, it is inherent to cross-sectional studies that a temporal link between the outcome and the exposure is lacking because both are examined at the same time, in our case at hospital admission. Nevertheless, we were able to detect the timing of symptom onset in both groups, which was between 6 and 7 days, thus reflecting the early phase of COVID-19 infection. Second, there are several scoring systems to predict severe COVID-19 (25), intensive care unit (ICU) admission (26), and mortality (13, 27, 28) based on the parameters available at hospital admission. Our database according to the RCT (3) had a low incidence of death and ICU admission, even though lungs were overweight. Thus, our study was not powered to detect variables collected at hospital admission that could identify mortality and ICU admission in patients with COVID-19. Third, we separated the excess lung weight into underweight and overweight values. We could have added normal weight lungs, however that would have reduced the statistical power because there were only a few cases and would require more complexity in the ROC analysis.

## Conclusion

The combinations of D-dimer/LDH/leukocytes, D-dimer/LDH/neutrophils, and D-dimer/LDH/leukocytes/neutrophils were the best predictors of overweight lungs in patients with COVID-19 pneumonia at hospital admission.

## Data availability statement

The original contributions presented in the study are included in the article/Supplementary material, further inquiries can be directed to the corresponding author.

## Ethics statement

This study adheres to the Declaration of Helsinki and the Brazilian National Commission for Research Ethics (CAAE:30662420.0.1001.0008), and individual Ethics Committees of all participating sites approved this study. The patients/participants provided their written informed consent to participate in this study.

## Author contributions

PLS participated in designing the study, collecting and analyzing the data, drafting the manuscript, and reviewing the manuscript. FFC contributed to designing the study, collecting and analyzing the data, and reviewing the manuscript. CMM contributed to analyzing the data and reviewing the manuscript. JH contributed to analyzing and interpreting the data, and reviewing the manuscript. SEG, YX, and MC contributed to analyzing and interpreting the data and drafting the manuscript. LB, PP, and PRMR participated in designing the study, contributed to analyzing and interpreting the data and drafting the manuscript. All authors approved the final version and agreed to be accountable for all aspects of the work ensuring that questions related to the accuracy or integrity of any part of the work are appropriately investigated and resolved.

## Funding

This study was supported by the Brazilian Council for Scientific and Technological Development (CNPq; 483005/2012-6), the Rio de Janeiro State Research Foundation (FAPERJ), the National Council for Scientific and Technological Development (CNPq; 483005/2012-6), Coordenação de Aperfeiçoamento de Pessoal de Nível Superior – Brasil (CAPES; Finance Code 001), and the Department of Science and Technology – Brazilian Ministry of Health (DECIT/MS).

## Conflict of interest

JH is cofounder and shareholder of OscillaVent, Inc. and a consultant for ZOLL Medical Corporation. CM is employed by AAC&T Research Consulting, LTDA.

The remaining authors declare that the research was conducted in the absence of any commercial or financial relationships that could be construed as a potential conflict of interest.

## Publisher’s note

All claims expressed in this article are solely those of the authors and do not necessarily represent those of their affiliated organizations, or those of the publisher, the editors and the reviewers. Any product that may be evaluated in this article, or claim that may be made by its manufacturer, is not guaranteed or endorsed by the publisher.

## Supplementary material

The Supplementary material for this article can be found online at: https://www.frontiersin.org/articles/10.3389/fmed.2023.1137784/full#supplementary-material

## Abbreviations

AUC: area under the curve
BMI: body mass index
CI: confidence interval
CT: computed tomography
Hb: hemoglobin
LDH: lactate dehydrogenase
RCT: randomized clinical trial
ROC: receiver operating characteristic
RT-PCR: reverse transcription polymerase chain reaction
SpO_2_: peripheral oxygen saturation
