# Supplementary material for: A specific combination of laboratory data is associated with overweight lungs in patients with COVID-19 pneumonia at hospital admission: secondary cross-sectional analysis of a randomized clinical trial
Source: Front Med (Lausanne). 2023 May 16;10:1137784. doi: 10.3389/fmed.2023.1137784 (PMC10228825; doi:10.3389/fmed.2023.1137784)
Supplement: Supplementary file 1 [file Data_Sheet_1.docx]

Supplementary Material

A specific combination of laboratory data is associated with overweight lungs in COVID-19 patients with pneumonia at hospital admission: secondary cross-sectional analysis of a randomized clinical trial

Pedro L. Silva, Fernanda F. Cruz, Camila M. Martins, Jacob Herrmann, Sarah E. Gerard, Yi Xin, Maurizio Cereda, Lorenzo Ball, Paolo Pelosi, Patricia R.M. Rocco

*** Correspondence:** Pedro L. Silva: pedroleme@biof.ufrj.br

# Supplementary figure

**
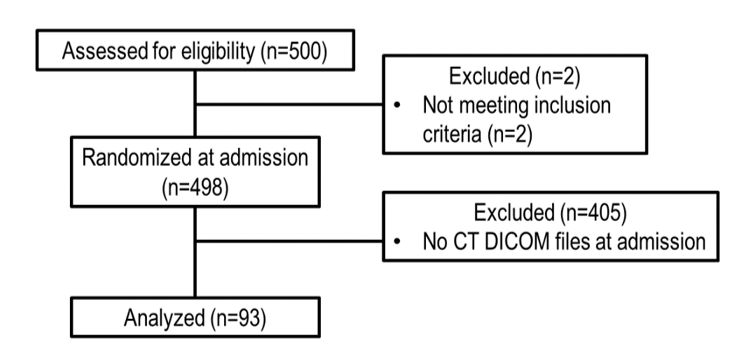
**

**Supplementary Figure 1.** Flowchart for inclusion of patients for the secondary cross-sectional analysis of the randomized clinical trial. Five hundred patients were assessed for eligibility. Two patients were excluded because they did not meet the inclusion criteria. At admission, 405 patients were excluded due to lack of CT DICOM files at hospital admission; 93 patients were analyzed.

**Supplementary Table 1.** Demographic parameters according to excess lung weight

| **Demographic parameters** | **Expected lung weight** | | | | ***P* value (chi-squared test)** |
| --- | --- | --- | --- | --- | --- |
|  | **Underweight** | | **Overweight** | |  |
|  | **N** | **%lin** | **N** | **%lin** |  |
| Age range |  |  |  |  |  |
| 18–40 years | 8 | 72.73 | 3 | 27.27 | 0.232 |
| 41–59 years | 17 | 43.59 | 22 | 56.41 |  |
| ≥60 years | 21 | 48.84 | 22 | 51.16 |  |
| Sex |  |  |  |  |  |
| Female | 17 | 62.96 | 10 | 37.04 | 0.151 |
| Male | 29 | 43.94 | 37 | 56.06 |  |
| Ethnicity |  |  |  |  |  |
| Asian | 0 | 0 | 1 | 100 | 0.681 |
| Black | 4 | 44.44 | 5 | 55.56 |  |
| White | 23 | 49.94 | 24 | 51.06 |  |
| Mixed | 19 | 52.78 | 17 | 47.22 |  |
| Body mass index |  |  |  |  |  |
| ≥30 kg/m^2^ | 9 | 33.33 | 18 | 66.67 | 0.09 |
| ≤29.9 kg/m^2^ | 36 | 55.38 | 29 | 44.62 |  |
| NI | 1 | 100 | 0 | 0 |  |

N, number of patients; %lin, percentage calculated by the total of the line as the denominator; NI, not informed.

**Supplemetary Table 2.** Clinical parameters according to excess lung weight

| **Clinical parameters** | **Lung expected weight** | | | | ***P* value (chi-squared test)** |
| --- | --- | --- | --- | --- | --- |
|  | **Underweight** | | **Overweight** | |  |
|  | **N** | **%lin** | **N** | **%lin** |  |
| Coexisting condition |  |  |  |  |  |
| Hypertension | 7 | 36.8 | 12 | 63.16 | 0.472 |
| Diabetes mellitus | 3 | 75 | 1 | 25 |  |
| Hypertension and diabetes mellitus | 3 | 37.5 | 5 | 62.5 |  |
| Chronic obstructive pulmonary disease | 0 | 0 | 1 | 100 |  |
| Asthma | 2 | 66.67 | 1 | 33.33 |  |
| Human immunodeficiency virus infection | 0 | 0 | 1 | 100 |  |
| None | 31 | 54.39 | 26 | 45.61 |  |
| Concomitant medications |  |  |  |  |  |
| Ivermectin | 0 | 0 | 0 | 0 | – |
| Hydroxychloroquine | 0 | 0 | 0 | 0 | – |
| Azitromycin | 2 | 40 | 3 | 60 | 1 |
| Angiotensin-II receptor antagonists | 3 | 33.33 | 6 | 66.67 | 0.504 |
| Angiotensin-converting enzyme inhibitors | 6 | 35.29 | 11 | 64.71 | 0.306 |
| Symptom at diagnosis |  |  |  |  |  |
| Dry cough | 44 | 51.16 | 42 | 48.84 | 0.449 |
| Productive cough | 1 | 50 | 1 | 50 | 1 |
| Sore throat | 3 | 75 | 1 | 25 | 0.594 |
| Shortness of breath | 32 | 43.84 | 41 | 56.16 | 0.069 |
| Diagnosis of SARS-CoV-2 infection |  |  |  |  |  |
| Negative | 4 | 100 | 0 | 0 | 0.12 |
| Positive | 42 | 47.19 | 47 | 52.81 |  |

N, number of patients; %lin, percentage calculated by the total of the line as the denominator.

**Supplementary Table 3.** Blood markers at hospital admission according to under and overweight lungs

| **Blood marker** | **Underweight (*n* = 46)** | **Overweight (*n* = 47)** | ***P* value (Mann-Whitney test)** |
| --- | --- | --- | --- |
| B-FGF | 291 [277] | 317 [345] | 0.226 |
| CTACK | 663 [944] | 820 [1243] | 0.150 |
| Eotaxin | 52 [296] | 82 [164] | 0.931 |
| G-CSF | 182 [220] | 222 [205] | 0.596 |
| GM-CSF | 7.1 [41.0] | 7.1 [41.2] | 0.878 |
| GRO-α | 801 [713] | 818 [905] | 0.589 |
| HGF | 861 [1664] | 1127 [1225] | 0.214 |
| IFN-α2 | 234 [136] | 361 [168] | 0.038 |
| IFN- γ | 52 [164] | 70 [219] | 0.611 |
| IL-10 | 73 [191] | 56 [206] | 0.686 |
| IL-12 (p40) | 1287 [1313] | 1469 [1627] | 0.532 |
| IL-12 (p70) | 30 [33] | 40 [50] | 0.252 |
| IL-13 | 162 [181] | 239 [308] | 0.855 |
| IL-15 | 80 [144] | 170 [160] | 0.422 |
| IL-16 | 362 [323] | 529 [349] | 0.153 |
| IL-17A | 75 [276] | 212 [282] | 0.365 |
| IL-18 | 359 [429] | 355 [365] | 0.589 |
| IL-1α | 233 [730] | 257 [721] | 0.316 |
| IL-1β | 65 [1230] | 241 [902] | 0.766 |
| IL-1ra | 2033 [1443] | 1867 [1629] | 0.943 |
| IL-2 | 57 [125] | 162,1 | 0.091 |
| IL-2Rα | 12810 [137259] | 7800000000 [810000000] | 0.400 |
| IL-3 | 6.32 [7.85] | 4.01 [6.20] | 0.321 |
| IL-4 | 6.7 [45] | 12.7 [27.1] | 0.863 |
| IL-5 | 355 [772] | 1153 [1949] | 0.309 |
| IL-6 | 8.29 [26] | 10.26 [21.01] | 0.888 |
| IL-7 | 917 [1682] | 669 [1286] | 0.452 |
| IL-8 | 142 [255] | 46 [80] | 0.222 |
| IL-9 | 137 [319] | 101 [95] | 0.428 |
| IP-10 | 1983 [3139] | 1795 [5529] | 0.482 |
| LIF | 7513 [4429] | 2739 [546] | 0.04 |
| M-CSF | 17 [293] | 2 [15] | 0.945 |
| MCP-1 | 149 [172] | 149 [160] | 0.487 |
| MCP-3 | 48 [173] | 144 [136] | 0.213 |
| MIF | 3864 [6381] | 2623 [1780] | 0.475 |
| MIG | 707 [470] | 671 [675] | 0.543 |
| MIP-1α | 7.4 [14.6] | 6.0 [10.3] | 0.969 |
| MIP-1β | 19.5 [10.4] | 21.8 [17.3] | 0.974 |
| PDGF-BB | 1820 [2927] | 1327 [3016] | 0.695 |
| RANTES | 420 [511] | 252 [417] | 0.130 |
| SCF | 65 [471] | 85 [570] | 0.505 |
| SCGF-β | 35936 [12662] | 33327 [19583] | 0.59 |
| SDF-1α | 48 [207] | 151 [627] | 0.489 |
| TNF-α | 31 [418] | 58 [138] | 0.642 |
| TNF-β | 242 [380] | 307 [1224] | 0.593 |
| TRAIL | 188 [740] | 84 [341] | 0.179 |
| β-NGF | 30 [50] | 86 [65] | 0.479 |

**Supplementary Table 4.** Receiver operation curve of blood marker data at hospital admission

| **Blood marker** | **AUC** | **95% confidence interval** | |
| --- | --- | --- | --- |
|  |  | **Inferior** | **Superior** |
| B-FGF | 0.662 | 0.421 | 0.903 |
| CTACK | 0.622 | 0.463 | 0.782 |
| Eotaxin | 0.508 | 0.325 | 0.692 |
| G-CSF | 0.571 | 0.320 | 0.822 |
| GM-CS | 0.485 | 0.303 | 0.667 |
| GRO-α | 0.565 | 0.337 | 0.794 |
| HGF | 0.606 | 0.440 | 0.772 |
| IFN-α2 | 0.764 | 0.547 | 0.982 |
| IFN-γ | 0.546 | 0.372 | 0.719 |
| IL-10 | 0.528 | 0.392 | 0.664 |
| IL-12 (p40) | 0.561 | 0.370 | 0.753 |
| IL-12 (p70) | 0.596 | 0.434 | 0.758 |
| IL-13 | 0.482 | 0.284 | 0.679 |
| IL-15 | 0.577 | 0.391 | 0.763 |
| IL-16 | 0.647 | 0.455 | 0.840 |
| IL-17 | 0.596 | 0.390 | 0.801 |
| IL-18 | 0.411 | 0.147 | 0.675 |
| IL-1α | 0.587 | 0.420 | 0.755 |
| IL-1β | 0.530 | 0.336 | 0.724 |
| IL-1ra | 0.507 | 0.338 | 0.675 |
| IL-2 | 0.659 | 0.483 | 0.836 |
| IL-2Ra | 0.833 | 0.371 | 1.000 |
| IL-3 | 0.587 | 0.409 | 0.765 |
| IL-4 | 0.516 | 0.345 | 0.686 |
| IL-5 | 0.659 | 0.355 | 0.962 |
| IL-6 | 0.510 | 0.370 | 0.651 |
| IL-7 | 0.580 | 0.365 | 0.794 |
| IL-8 | 0.629 | 0.426 | 0.831 |
| IL-9 | 0.591 | 0.371 | 0.811 |
| IP-10 | 0.551 | 0.405 | 0.698 |
| LIF | 0.821 | 0.536 | 1.000 |
| M-CSF | 0.509 | 0.299 | 0.719 |
| MCP-1 | 0.439 | 0.266 | 0.612 |
| MCP-3 | 0.647 | 0.427 | 0.868 |
| MIF | 0.606 | 0.329 | 0.884 |
| MIG | 0.560 | 0.371 | 0.749 |
| MIP-1α | 0.504 | 0.339 | 0.669 |
| MIP-1β | 0.505 | 0.339 | 0.669 |
| PDGF-BB | 0.534 | 0.339 | 0.669 |
| RANTES | 0.630 | 0.464 | 0.796 |
| SCF | 0.567 | 0.374 | 0.761 |
| SCGF-β | 0.600 | 0.341 | 0.859 |
| SDF-1α | 0.576 | 0.358 | 0.793 |
| TNF-α | 0.545 | 0.351 | 0.738 |
| TNF-β | 0.667 | 0.168 | 1.000 |
| TRAIL | 0.648 | 0.447 | 0.849 |
| β-NGF | 0.667 | 0.159 | 1.000 |
